# IMPACT OF BODY MASS INDEX ON PERIOPERATIVE OUTCOMES FOR COMPLEX HIATUS HERNIA BY VIDEOLAPAROSCOPY

**DOI:** 10.1590/0102-672020220002e1672

**Published:** 2022-09-09

**Authors:** Renato Abrantes Luna, Eduardo Mesquita Peixoto, Cecília Ferreira de Araújo Carvalho, Luciane de Souza Velasque

**Affiliations:** 1 Universidade Federal do Estado do Rio de Janeiro, Faculty of Medicine and Surgery, Federal Hospital Rio de Janeiro State – Rio de Janeiro (RJ), Brazil; 2 Fiocruz National Institute of Infectious Diseases, Rio de Janeiro (RJ), Brazil; 3 Federal Hospital Rio de Janeiro State, Rio de Janeiro (RJ) – Brazil; 4 Universidade Federal do Estado do Rio de Janeiro, Biostatistics Department, Rio de Janeiro (RJ), Brazil.

**Keywords:** Body Mass Index, Obesity, Hiatal, Hernia, Gastroesophageal Reflux, Laparoscopy, Postoperative Complications, Índice de Massa Corporal, Obesidade, Hernia Hiatal, Refluxo Gastroesofágico, Laparoscopia, Complicações Pós-Operatórias

## Abstract

**BACKGROUND:**

The influence of body mass index on perioperative complications of hiatal hernia surgery is controversial in the surgical literature.

**AIMS:**

The aim of this study was to evaluate the influence of body mass index on perioperative complications and associated risk factors for its occurrence.

**METHODS:**

Two groups were compared on the basis of body mass index: group A with body mass index <32 kg/m^2^ and group B with body mass index ³32 kg/m^2^. A multivariate analysis was carried out to identify independent predictors for complications. Complications were classified based on the Clavien-Dindo score.

**RESULTS:**

A total of 49 patients were included in this study, with 30 in group A and 19 in group B. The groups were compared based on factors, such as age, Charlson Comorbidity Index, surgical techniques used, type and location of hiatal hernia, and length of stay. Findings showed that 70% of patients had complex hiatal hernia. In addition, 14 complications also occurred: 7 pleuropulmonary and 7 requiring reoperation. From the seven reoperated, there were three recurrences, two gastrointestinal fistulas, one diaphragmatic hernia, and one incisional hernia. Complications were similar in both the groups, with type IV hiatal hernia being the only independent predictor.

**CONCLUSIONS:**

Body mass index does not affect perioperative complications in anti-reflux surgery and type IV hiatal hernia is an independent predictor of its occurrence.

## RESUMO

**RACIONAL:**

O impacto do índice de massa corpórea nos resultados da cirurgia de hérnia de hiato é controverso na literatura.

**OBJETIVOS:**

avaliar o impacto do índice de massa corpórea nas complicações perioperatórias em pacientes submetidos a cirurgia de hérnia hiatal, e seus possíveis preditores.

**MÉTODOS:**

análise retrospectiva 49 pacientes submetidos a tratamento cirúrgico de hérnias hiatais complexas por videolaparoscopia, divididos em dois grupos pelo índice de massa corpórea (grupo A<32kg/m2 – 30 pacientes e grupo B ³32 kg/m2 — 19 pacientes) e comparados quanto suas características e complicações. A análise multivariada foi aplicada para avaliar as variáveis preditoras independentes de complicações. As complicações foram classificadas conforme Clavien Dindo.

**RESULTADOS:**

Os grupos foram similares conforme a idade, índice de comorbidade de Charlson, técnica operatória empregada, tipo de hérnia de hiato, área do hiato esofageano, e tempo de internação pós-operatória. Setenta por cento dos pacientes possuíam hérnias de hiato complexas (gigantes ou recidivadas). Catorze complicações foram observadas: 7 pleuro pulmonares e 7 necessitando reoperação, sendo destas 3 recidivas, 2 fístulas digestivas, 1 hérnia diafragmática e 1 hérnia incisional. As complicações foram semelhantes em ambos os grupos, e a hérnia de hiato tipo IV foi o único preditor independente.

**CONCLUSÕES:**

O índice de massa corpórea não influencia nos resultados perioperatórios e a hérnia de hiato tipo IV é o único preditor independente de complicações.

## INTRODUCTION

Reflux disease is highly prevalent in modern society and influenced by the global epidemic of obesity^5^. The association between these conditions is not uncommon^10^.

Laparoscopic fundoplication is the surgical treatment of choice for symptomatic hiatal hernia, with good long-term clinical results and safety characteristics. One of the confirmed risk factors for perioperative complications of hiatal hernia surgery is an elevated body mass index (BMI)^8,14^; however, there is no consensus on this issue, with contradictory reports on literature.

The aim of this study was to evaluate the influence of BMI on perioperative complications related to symptomatic hiatal hernia surgery and assess possible risk factors associated with it.

## METHODS

This is a retrospective analysis of a prospectively maintained database at the General Surgery Department of Servidores do Estado do Rio de Janeiro Hospital. This cohort included all patients who underwent laparoscopic hiatal hernia surgery and had anthropometric measures for BMI calculation. Two patients’ cohorts based on BMI were created: group A with BMI <32 kg/m^2^ and group B with BMI ≥32 kg/m^2^. These groups were compared based on factors, such as symptoms, age, Charlson Comorbidity Index^6^ (CCI), type and location of hiatal hernia, and hospital length of stay (LOS). Next, nonstandard techniques like Collis gastroplasty, gastropexy, or hiatal mesh augmentation, and conversion to laparotomy were noted. Later, an attending surgeon examined the patients, and extracted perioperative complications from chart review. The hiatal location was analyzed and patients were divided into two groups: those with location <10 cm^2^ and those with ≥10 cm^2^. Complications were classified based on the Clavien-Dindo score^9^.

Symptoms were generally defined in three groups: reflux, when heartburn and regurgitation predominate; obstructive, when dysphagia and chest pain were the main complains; and mixed, when an association between heartburn, regurgitation, and dysphagia occurred.

Gastroesophageal reflux disease (GERD) was diagnosed when LA grade B esophagitis, Barrett's esophagus, or peptic stenosis present at upper endoscopy, or a pathological pH study was observed. A CT scan or an esophagram was used selectively when patients had obstructive symptoms. All patients were confirmed with hiatal hernia.

Hiatal hernias were classified as follows^4^:

- Type I: sliding hiatal hernia;
- Type II: paraesophageal hiatal hernia or rolling hiatal hernia;
- Type III: mixed hiatal hernia, with a sliding component associated with a rolling fundus;
- Type IV: hiatal hernia associated with a herniation with other organ.

Collis wedge gastroplasty was applied under the surgeon discretion when a 3-cm reduction of the esophagogastric junction (EGJ) under the diaphragm was not achieved after deep hiatal esophageal dissection, according to Hunter and colleagues^18^. In short, after complete dissection of the hiatal hernia and short gastric vessel division, a 48Fr bougie was inserted. Sequential staples firing directed from the greater curvature toward the bougie, to a point 3 cm below the left crura. After the dilator is reached, vertical staples firing toward the His angle is achieved, with a resection of a small triangle of the fundus.

The stomach was fixed to the anterior abdominal wall in two ways: a formal tubular gastrostomy with a 24Fr catheter fixed with four transfacial stitches or by just two nonabsorbable simple stitches between the greater curvature and the anterior abdominal wall.

When mesh augmentation was applied, its fixation was performed with simple stitches until good apposition to the crura was obtained. All polypropylene meshes were covered by omentum, to protect EGJ from erosion^3^.

This research was approved by the Local Ethics Committee under the number 133171/2020 on December 14, 2020, protocol CEP 000.660, and consent was waived owing to the retrospective nature and identification of the data.

The Student's t-test and Wilcoxon test were used for parametric and nonparametric data, while chi-square and Fischer's exact tests were used for qualitative variables. A univariate analysis and a stepwise selection method were performed for a multivariate model, looking for independent factors associated with complications.

## RESULTS

From an initial cohort of 71 patients, complete data were available for 49 patients. Out of 49 patients, 30 with BMI <32 kg/m^2^ were assigned to GROUP A and 19 with BMI ≥32 kg/m^2^ were assigned to GROUP B. In all, 35 (71%) patients had complex hiatal hernias (paraesophageal type III with more than 50% of the stomach herniated or type IV) or recurrent. Reflux symptoms (heartburn and regurgitation) were observed in 27 patients, obstructive symptoms (dysphagia and chest pain) in 17, and a mix of both reflux and obstructive in 5. All patients had a standard crural repair and a Toupet or Nissen fundoplication. Adjuvant techniques were applied when needed.

Mesh augmentation was performed in 16 patients, gastropexy in 15, and a Collis gastroplasty in 5. In fact, 90% of operations were performed by two surgeons in an attempt to standardize the procedures. There was one conversion to open surgery in each group. The median length of stay was 2 days in each group. Hiatal location was measured in 16 (53%) of patients in group A and in 7 (37%) patients of group B (mean of 9.66 and 8.29 cm^2^, respectively).

Upper endoscopy was performed in all patients before surgery, while a pH monitoring was done in 17 patients who had hiatal hernia and reflux symptoms; however, upper endoscopy did not fulfill the criteria for the diagnosis of GERD.

Both groups did not show any difference in age, Charlson Comorbidity Index (CCI), surgical techniques used, type and location of hiatal hernia, or surgeon experience (Table 1).

**Table 1:** Group's characteristics.

		BMI <32 (n=30)	BMI ≥32 (n=19)	p-value
BMI (mean, SD)		27.6 (2.4)	35.3 (3.2)	<0.001&
Age (median, IQR)		63 (60.9–69.7)	60.5 (52–65)	0.058*
CCI (IQR)		2 (2–3)	2 (1–2.5)	0.297*
Hiatal hernia type				0.918$
	Type I	8	6	
	Type II	1	0	
	Type III	14	7	
	Type IV	3	3	
	Recurrent	4	3	
Mesh				0.664$
	Polypropylene	5	5	
	PGA/TMC+	4	2	
Gastropexy				0.786$
	Gastrostomy	2	1	
	Gastropexy	6	6	
Collis		3	2	0.486$
Surgeon				0.35$
	A	18	9	
	B	8	9	
	Other	4	1	
Hiatal location (n)		9.66 (16)	8.29 (7)	0.281&
LOS (IQR)		2 (2–3)	2 (2–3)	0.543*
Complication		9	5	0.781#

* Wilcoxon test;

$ Fischer's exact test;

# Chi-square test;

& Student's t-test; PGA: polyglycolic acid; TMC: trimethylene carbonate;

+ 67% polyglycolic acid and 33% trimethylene carbonate; BMI: body mass index; SD: standard deviation; CCI: consumer confidence index; LOS: hospital length of stay; IQR: interquartile range.

Perioperative complications were similar in both groups, with no influence of BMI (five patients with BMI ³32 and nine patients with BMI <32) (Table 2).

**Table 2:** Surgical complications.

	BMI <32	BMI ≥32	Clavien-Dindo
Pulmonary	5	2	
Pleural opening	3	2	1/3a*
Atelectasis	1	0	1
PTE	1	0	4b
Recurrence	1	2	
Diaphragmatic hernia	1	0	3b
Fistula	2	0	
Esophageal	1	0	3b
Small bowel	1	0	4b
Incisional hernia	0	1	3b
Total	9	5	

* One patient needed chest drainage; BMI: body mass index; 3a,3b and 4b: Clavien-Dindo score; PTE: pulmonary thromboembolism.

Pulmonary diseases were the most frequent complications, with five unplanned pleurotomies, one atelectasis needing Continuous Positive Airway Pressure (CPAP) and respiratory physical therapy, and one deep vein thrombosis with pulmonary thromboembolism (PTE). Only one patient needed chest tube drainage. Seven patients had complications that required additional surgery. Two of them had early hernia recurrences and underwent reoperation within 48 h. One patient developed an esophageal fistula which was diagnosed and reoperated on 7 days after index operation. One patient with a history of multiple abdominal surgeries developed a small bowel fistula related to abdominal cavity access. One patient presented with a hiatal hernia recurrence 3 months post-op. Another patient presented with a small diaphragmatic hernia close to the left crus identified during the workup of postoperative pain, and one patient developed a trocar site incisional hernia.

All patients were successfully treated, and no mortality was observed. One conversion to open surgery was observed in each group. A single conversion to open was necessitated by inability to progress related to multiple abdominal procedures. The second case was related to an equipment problem that prevented the surgery to continue safely laparoscopically.

Once BMI was not shown to influence the outcomes, univariate and multivariate analyses were performed looking for predictors of complications. First, we observed that polyglycolic acid/trimethylene carbonate (PGA/TMC) mesh (p=0.049, OR=7.44) and type IV hernia (p=0.013, OR=29.81) were associated with complications. In a more detailed analysis, it was observed that PGA/TMC mesh-associated complications were related to pleural opening. Considering that pleural opening is related to the hiatal dissection and not to mesh implantation, a second model was run, considering only complications that need reoperations. In this model, the use of mesh was no longer correlated with complications, with the type IV hernia still significant (p=0.013, OR=22.98).

## DISCUSSION

Our study was not able to show an influence of BMI on perioperative outcomes. The results did show that the presence of a type IV hiatal hernia has an independent influence on outcome. It is not clear which BMI cutoff point discriminates higher complications or failure of anti-reflux surgery. Our option was to use BMI of 32, because patients with BMI >35 are ideally treated with Roux-en-Y gastroplasty.

The literature is controversial about BMI influence on hiatal hernia surgery outcomes, with divergent results^7,13,16,17^. Our results demonstrate, at least short term, that there is no influence of BMI on postoperative complications, when a BMI cutoff point of 32 is used. Overall, we can suggest that short-term results are similar; however, long-term BMI may adversely affect anti-reflux surgery outcomes, according to recent meta-analyses available^1,2^.

About 70% of our patients had complex hiatal hernias, meaning giant paraesophageal or recurrent hernias. This resulted in the need for crural mesh augmentation in 32% of patients, gastropexy in 31%, and Collis gastroplasty in 10%. The application of these techniques was based on surgeon judgment. The limiting of these operations to those conducted by two attending surgeons (90% of the cases) was an attempt to decrease the influence of surgeon bias between cases. During the study period, hiatal surface area of 10 cm^2^ became our threshold for mesh utilization^11^, but initially its use was based on surgeon subjective judgment. The BMI alone was not an indication for mesh as proposed by some authors^15^. Given that the use of advanced techniques was similar in both groups, it is postulated that BMI did not interfere or prevent their utilization.

It is of interest to note that only type IV hiatal hernia was an independent predictor of postoperative complications. Somewhat unexpected for us was the correlation of the biosynthetic absorbable mesh with complications in our first model. Given that pleural opening and mesh application happen at different stages of the procedure, we could not correlate its use with the increased frequency of complications. When we considered only complications that lead to reoperations and correlated with hiatal manipulation, type IV hernia was still an independent factor.

Current literature shows complications between 3 and 45%, with a 30-day reoperation between 1.6 and 4.9%^12^. Our results correlate positively with the literature, with Clavien-Dindo score >3 complications happening in 18% (9/49) and 30-day reoperation rate of 8% (4/49).

The discussion of how BMI influences outcomes in anti-reflux surgery is still pertinent. In environment with limited access to bariatric surgery, treatment of hiatal hernia surgery is often the final option for patients with low quality of life. It is important to realize that most of our patients had complex hernias, which would be presumed to increase surgical complications when coupled to higher BMI. This was not the case.

This study is limited by relatively small numbers and the ability to gather additional data points retrospectively. This resulted in the inclusion of only 49 out of a total 71 patients available. It is unclear about the influence that the additional patients may have had on the data analysis. Also, the lack of long-term follow-up limits our conclusions to the perioperative period.

## CONCLUSION

BMI has no impact on perioperative complications after anti-reflux surgery, even when complex hiatal hernias are treated. The presence of type IV hiatal hernia independently predicts perioperative complications. The use of adjuvant techniques is possible in patients with higher BMI with good results.
